# Linkage to care and treatment among men with reactive HIV self-tests after workplace-based testing in Uganda: A qualitative study

**DOI:** 10.3389/fpubh.2022.650719

**Published:** 2022-10-12

**Authors:** Patience A. Muwanguzi, LaRon E. Nelson, Tom D. Ngabirano, Noah Kiwanuka, Charles Peter Osingada, Nelson K. Sewankambo

**Affiliations:** ^1^School of Health Sciences, College of Health Sciences, Makerere University, Kampala, Uganda; ^2^Yale School of Nursing, Yale University, New Haven, CT, United States; ^3^School of Public Health, College of Health Sciences, Makerere University, Kampala, Uganda; ^4^School of Medicine, College of Health Sciences, Makerere University, Kampala, Uganda

**Keywords:** HIV self-testing, men, Sub-Saharan Africa, linkage to care, workplace

## Abstract

**Introduction:**

HIV self-testing at workplaces has the potential to reach men at risk of HIV infection with lower access to HIV testing services. While several studies have reported high uptake of HIV self-testing, linkage to HIV care following a positive result remains a challenge. This study, therefore, explored the motivators for and barriers to linkage to HIV care and treatment among men who returned positive results following workplace-based HIV self-testing.

**Methods:**

A qualitative descriptive study, among men in private security services in Kampala district, Uganda. The men were eligible to participate if they were aged 18 to 60 years and had worked at the company for more than 6 months. Following HIV self-testing, participants with reactive (positive) self-test results were purposively sampled and engaged in key informant interviews. Inductive content analysis was employed to identify the motivators and barriers to the men's linkage to HIV treatment and care.

**Results:**

Overall, 12 men participated in the study, of whom 9 (75%) were security guards, and the rest held management positions. The motivators for linkage to care coalesced under five categories. (i) Communication (open communication, phone reminders, consistent communication) (ii) Navigating health facility systems and processes (enabling health facility environment, easy access to health care, employing ART clinic counselors as part of the study team, health workers) (iii) Linkage support (linkage companions, referral forms, linkage facilitation, individualized linkage plan, pre-arranged clinic appointments) (iv) Psychosocial support (counseling sessions, family support, online and social media support, peer support) (v) workplace environment (employer's support, work schedules and policies). The barriers to linkage to HIV care included (i) Inflexible work schedules, (ii) Far distances to travel to access ART (iii) mandatory work transfers, (iv) disruptive effects of the COVID-19 pandemic, (v) Denial of HIV-positive results and (vi) fear of stigma and discrimination at health facilities.

**Conclusion:**

The findings suggest the need for innovative interventions to facilitate regular follow-up and open communication with workplace-based HIV self-testers, to improve linkage to HIV care and treatment. Furthermore, initiating linkage plans during pre-test counseling and working in collaboration with health facilities and clinics may improve linkage to care.

## Introduction

An estimated 38 million individuals are living with HIV globally (1). Women have historically been more likely than men to take an HIV test or link to care (2). In 2019, the Joint United Nations Programme on HIV/AIDS (UNAIDS) reported that 79% of women living with HIV in Uganda were on HIV treatment, compared to 63% of men (3). Several reasons have been suggested for why men may not engage in HIV testing. Evidence suggests that HIV self-testing (HIVST) may overcome the hindrances that have been reported to the uptake of HIV testing services, including stigma and lack of privacy and confidentiality (4). HIV self-testing at workplaces can further reach men with limited access to HIV testing services and yet are at risk of HIV infection (5). Some of the enablers for the uptake of HIVST include; the ease of accessibility of the self-test kits (6), and the perceived convenience because one can take the test anywhere and at any time (7). Additionally, HIVST overcomes stigma and discrimination, and challenges faced at health facilities since the test is taken privately and independently (8). Furthermore, HIVST assures greater confidentiality of test results than at the health facility (9). Several studies have reported challenges with the uptake of HIVST including concern about the unreliability of tests and low literacy levels about HIVST (10), the anxiety of the repercussions of a reactive test result and the unaffordable cost of the self-test kits (7). Additional concerns raised regarding HIVST include the potential for coercion into taking a test (6). Several studies report recurring challenges in ascertaining and confirming HIVST results (11), and linking individuals to HIV treatment and care following a reactive self-test result (12). While studies have reported high uptake of HIVST in other populations (13–17), linkage to care and measurement of linkage following HIV self-testing remains a challenge (18). We implemented a qualitative study embedded in a clinical trial, to inform the design of future workplace HIV self-testing linkage initiatives, (ClinicalTrials.gov Identifier: NCT04164433) (19). This study explored the motivators for and barriers to linkage to HIV care and treatment among men who received positive self-test results following HIVST in workplace settings.

## Methods

### Study setting

The descriptive qualitative study was conducted as part of the **W**orkplace-based H**I**V **Se**lf-testing among **Men** (WISe-Men), Cluster randomized trial (Clinical trials.gov ID NCT04164433) (20). In the WISe-Men trial, men working in private security services in two Ugandan districts were offered HIV self-testing or standard HIV testing services at their workplaces. This qualitative descriptive study employing in-depth interviews took place between April and June 2020, at private security companies employing at least 50 men each. We conducted the qualitative study in Kampala district only because this was the trial arm that received HIV self-testing.

### Research team and reflexivity

PAM and LEN have expertise in qualitative health research, and all the research team members are health researchers at varying levels of research experience. This qualitative study is nested in the WISe-Men clinical trial; therefore, the research team had an existing relationship with the participants. The participants knew about the overall study and were familiar with the overall and specific objectives of the study.

### Study participants and selection

Two months after receiving the HIVST intervention, 20 participants with reactive (positive) HIV self-test results were purposively selected and approached and their consent was sought for participation in this sub-study. Two (2) declined, while one (1) potential participant had a very unstable telephone network which made data collection difficult. Therefore, 17 were eligible for participation in the study, however, enrollment stopped at 12 participants when no new information was obtained from the interviews (data saturation).

Men were eligible to participate if they were aged 18–60 years and had worked at the company for more than 6 months. The men were engaged in in-depth interviews until a point of data saturation was attained, where no new information emerged from the interactions.

### Ethical considerations

Ethical approval was granted by both Makerere University School of Health Sciences Research Ethics Committee (SHS-REC) (Ref. 2018-054), and the Uganda National Council of Science and Technology (UNCST) (Ref. HS 2672). Furthermore, administrative clearance was obtained from the responsible personnel officer at the private security company. Each participant gave individual written consent before enrolment in the WISe-Men trial. Since we conducted phone interviews, the men sent a text message as written consent and gave verbal consent at the start of the interview. Involvement in the study was voluntary and there were no repercussions for non-participation.

### In-depth interviews

One-time phone interviews were conducted with the participants during the Coronavirus disease (COVID-19) lockdown period in Uganda (21). Two trained research assistants and PAM made all the phone calls from a private room, on speakerphone and participants consented to an audio recording of the interview. Each interview lasted 45 min to 1 h and employed a semi-structured and open-ended interview guide. The guide was piloted by three men from one security company and their data is not included as part of this study. The questions in the guide sought information regarding what motivated, delayed, or prohibited their linkage to treatment and care, the challenges faced in accessing posttest services, and the men's perceptions on how linkage to treatment and care may be optimized. Field notes were made during each interview. Data collection stopped when no new information (saturation) emerged from the interviews.

### Data analysis

The data were transcribed verbatim by PAM and the two research assistants who were involved in data collection. The transcripts of the audio recordings were analyzed in NVivo 12 pro (QSR International) using qualitative content analysis following the procedure by Elo and Kyngas (22). Initially, two team members (PAM and TDN) reviewed the transcripts while continually listening to the audio recordings to ensure that all the information was captured accurately. The transcripts were then read in their entirety to gain immersion into the data and obtain a sense of the whole. The pair undertook the open coding process separately to identify meaningful phrases and codes, and then convened to attain a consensus. Any disagreements that arose were settled by a third member of the study team. The coding team iteratively placed the codes into groups according to the similarity of patterns to form subcategories and then categories.

To ensure trustworthiness and the quality of the data, a sample of the study participants reviewed the categories and subcategories. Interview notes were recorded in the principal researcher's reflective journal for confirmability. Additionally, the degree of congruence attained between the two individuals during analysis provided data accuracy and meaning. Furthermore, prolonged participant engagement during the interviews allowed each participant enough time to express his views. For transferability, this paper provides a rich description of the participants' narratives (23, 24). This paper is guided by the Consolidated criteria for Reporting Qualitative research (25).

## Results

### Participants' characteristics

Therefore, 12 men participated in the study, of whom 9 (75%) were security guards with the rest in management positions, 4 (33.3%) were aged 26–35 years, 8 (66.7%) were married and 7 (58.3%) had completed secondary education Supplementary Table 1.

### Motivators for linkage to care and treatment

The motivators for linkage to care and treatment coalesced around five primary topics: communication, navigating the health facility systems and processes, linkage support, psychosocial support, and workplace environment. The Participant quotations are presented to illustrate the categories and sub-categories. Supplementary Table 2 provides a summary of the coding tree for the motivators for linkage to care.

#### Communication

Communication emerged as a common thread in all the participant's interviews. Many felt that the open channels of communication and availability of the health workers to respond to their queries motivated their linkage to care. Some also suggested that it was the consistent communication that facilitated this process. The fact that their health providers did not give up on them was critical.

**Phone reminders**. Many reported that the phone reminders, text messages and the information discussed during the call played a critical role in the decision to link to HIV treatment and care.

*I told her* [the nurse] *that I was available to talk only on Saturday evenings. She called me every Saturday to check on me and sometimes it was another person, but the message was the same. When I asked her many questions, she would send me information via WhatsApp after the discussion so that I could do further reading. I finally went after 6 weeks*. (P3, 33 years)

**Consistent and regular communication**. Initially, some participants found it hard to accept their results, however, the consistent calls and counseling from the research team helped them to come to terms with the result and seek further testing and care.

*Before the test, the health workers asked us for our phone numbers and permission to call us after we had taken the HIV kit. Two days later, she called me to find out if I had taken the test. I had but was not yet ready to talk. After about a week, she called me again and I was feeling better, so I shared my results. She counseled me and requested permission to make a weekly call. She called me consistently and after 2 months, I was ready to go to the hospital*. (P9, 46 years)

**Open channels of communication**. The men reported that the health workers kept the channels of communication open which allowed them to seek answers to all their worries and questions. They did not feel pressured to go for further care and felt that they were always in control of their decision.

*I always felt in control. The nurses did not pressure me at all to go to the hospital. They gave me a special number that I could contact at any time if I had any questions. At the start, I called them every day, but they were very understanding. I liked that openness from them even though they did not know me*. (P10, 51 years)

#### Navigating the health facility systems and processes

Several studies have previously reported the challenges of navigating health facility processes including long lines and stigma as major reasons why men do not link to care and treatment. In this case, the men reported that the effort that the health workers put in to ensure a smooth transition at the health facility was largely responsible for their linkage to care.

**Inclusion of health facility staff on the research team**. Several participants found it easy to navigate the health facility, because some of the staff at the hospital were familiar, as they had participated in the workplace testing. This helped with establishing trust and strengthening linkage and retention in care.

*Some of the staff working at the hospital, were also part of the group that did the testing for us at the office. So, it was easy when I went for treatment because I had already created rapport with the health workers and felt that they were trustworthy*. (P11, 52 years)

**Enabling health facility environment**. For some participants, the non-stigmatizing environment at the hospital facilitated their linkage to care and treatment.

*No one was looking at me badly, it seemed like none of the other patients cared why I was there. People were receiving treatment and I felt that this was just another illness with its clinic. It is not what I expected at all. I even told my colleague who was hesitating to go and when he went, it was a similar experience*. (P01, 20 years)

**Easy access to care**. Limited time to access HIV services was a big concern for many of the participants. Therefore, the short time spent at the facility was a motivator for linkage to care.

*The referral chit* [form] *was helpful because it had all the information that was required at the hospital. This made the process so much faster for us. All of us who had those pink forms were seen immediately*. I got another test, did some other blood tests, and was started on my HIV medication very quickly. I did not even have to take a sick day because I went home and rested enough to work the night shift. (P12, 36 years)

**Trustworthiness of health workers. One** of the fears expressed by the men before the test was the potential for breach of confidentiality by the health workers, particularly to their employers. Therefore, when their employers did not mention that they had positive results or treat them any differently, then they felt that they could trust the health workers and seek further care.

*I was concerned that the nurses were going to tell my boss that I am HIV positive, and I would have denied it. But I went to work for 3 more weeks, and my boss did not say anything. Therefore, it meant that even if I were to visit the hospital, my information would still be safe. So, I went to the hospital about three and a half weeks after the test because I felt that I could trust them*. (P04, 35 years)

### Linkage support

The participants appreciated the support that they received in linking to the health facilities. This was in the form of a pre-planned linkage plan, transport facilitation to the hospital, referral forms and the pre-arranged clinic appointments. For many participants, this was the key motivator for linking to further management.

**Individualized linkage plan**. Several participants appreciated the creation of a linkage plan during the pre-test counseling session. This plan gave them a clear course of action when they received reactive self-test results.

*Before receiving the test kit, I met with the counselor and the nurse. We agreed on three possible hospitals I would go to in the event of positive results. Whether I wanted peer support, someone to go with me to the health facility, who I would share my results with and if I was comfortable with the phone to follow up. When I got my results, I was not so confused because we had talked about all this before, and the plan of action was clear*. (P03, 33 years)

**Linkage facilitation**. The participants verbalized that the facilitation they received enabled them to link to care. The facilitation was in the form of organizing travel for the men to access the health facilities as well as travel vouchers.

*During this time, I had moved upcountry because of the COVID-19 lockdown. My counselor called me and when I told her that I couldn't access the health facility, she organized with the people at the hospital close to my home and they sent a car to come for me. Because of this, I was still able to reach the facility and start the treatment. When I came back to my workstation, the counselor took me personally and I was able to transfer my care*. (P11, 52 years)

**Referral forms**. We designed referral forms with input from health workers responsible for registration at health facilities. The participants reported that these forms made it possible for them to receive care much faster since the health workers already knew about to expect these slips.

*When I went to the health center, the lady where we go for registration welcomed me and when I showed her my referral slip, she quickly directed me to where I needed to go and did not ask me so many questions because some information was already on the slip*. (P07, 36 years)

**Study team members act as linkage companions**. Some participants who had initial difficulty with linking to care received active linkage support from the study team members. This support was a strong enabler of linkage to care.

*The lady* [study nurse] *called me to find out how I was doing, and I told her that I was worried about going to the hospital. I told her all my fears and she proposed that I should meet her at the hospital. We went together to the ART clinic; she went with me throughout the entire hospital journey. I appreciate the help she gave me during my first hospital visit*. (P02, 25 years)

**Pre-arranged clinic appointments**. Participants expressed that the ability to make appointments at the clinic was one of the motivating factors for linking to care.

*One of my biggest worries was about the line at the health facility because the time I spend at work it is extremely hard for me to go to the hospital and take a day off. Our counselor told us to meet her on Tuesday at the hospital and everything went extremely fast. Can you believe I got everything done during my lunch break? It was a pleasant surprise*. (P06, 35 years)

#### Psychosocial support

Several participants suggested that they would not have been linked to care if they did not receive counseling and online support from the health workers. Additionally, some men felt emboldened to seek further care because they had the support of their family, and peers.

**Counseling sessions**. Some participants did not expect to receive reactive HIVST results and could not cope with the diagnosis. A few suggested that they owed their linkage to further care, to the support and the sessions that they received from their counselors.

*When I got the result, I was devastated. I went home and could not face my wife. I was not sure where this disease came from. I was so bitter and was going to do something very harmful either to myself or to her. The counselor called me the next day because I had told her that I was available on Fridays and from our talk she got concerned. She asked me to make time and go to the health facility. We met and discussed the diagnosis and after about seven in-person sessions, I was able to accept this and go to the hospital. This was about 8 weeks later*. (P08, 40 years)

**Online and social media support**. During the pre-test counseling session, some participants requested online follow-up support, including the utilization of different social media sites and applications. The regular communication with the counselor helped them to decide to seek HIV care.

*I have a smartphone so she* [the study counselor] *asked me how I preferred to be contacted after the test. I opted for WhatsApp messages because of privacy. She always started the chat with a code question and when I responded with the answer, then she knew it was me. This chat was helpful for me, and that constant open communication is what helped me to go to the hospital after I received the bad news* [reactive self-test]. (P05, 35 years)

**Family linkage support**. Several participants preferred to go for further testing and antiretroviral (ART) initiation closer to home, with their spouses and other family members. This support to link as a family encouraged the men to seek HIV care.

*The support from my wife and family encouraged me to go to the health facility when I tested positive. I work in Kampala, but my family lives in another district, so I traveled there and got treatment because I could get support from home*. (P10, 51 years)

*They provided my transport, and it was good for me because I wanted to go for further testing and treatment with my wife. This gave me peace of mind*. (P10, 51 years)

**Peer-support**. Some participants expressed that peer support was a strong influence on their actions following the reactive self-test result.

*The counselor asked me if I was willing to support other people who were struggling with their diagnosis. I agreed and she sent me to two other people who tested positive. We formed a small accountability group, and we follow up with each other. This has helped us to continue with our treatment and to have people to talk to*. (P07, 36 years)

### Workplace environment

The HIV testing intervention was conducted at the men's workplace. Therefore, the workplace environment was a key factor in their decision to seek care and treatment. The environment included the presence or absence of the employer's support and the work schedules and policies.

**Employers' support**. Support from employers was given in different forms including time off and mitigating potential stigma and discrimination at the workplace. One participant expressed his gratitude to their employer as follows:

*Since we tested with our supervisors, they gave me the support I needed. They also did not disclose my status to my other colleagues, because I have not seen any change in how my workmates interact with me*. (P12, 36 years)

**Workplace schedules and policies**. Some participants expressed that they were able to discuss with the employers or responsible managers and were given time off to go to the health facility. They suggested that this had only been possible because the testing had taken place at the workplace. It may have been different if the testing was in a health facility or elsewhere. They also had some workplace policies that offer punitive measures for people who discriminate against others for whatever reason. Therefore, they felt comfortable going for further care because they had support at the workplace.

*Testing at the workplace made it easier for me to get time off to go for further treatment. If I had taken the test elsewhere, it would have been complicated. So, this means that testing at the workplace is helpful* (P08, 40 years)

*We have a policy here where people are not supposed to discriminate against others for whatever reason maybe disability etc. Someone can even lose their job. Therefore, I was confident that nothing would happen to me, and I was able to access care after 2 weeks*. (P07, 36 years)

### Barriers to linkage to care and treatment

These are presented under four categories: workplace-related barriers, socio-economic barriers, health facility-related barriers and personal/individual factors. Supplementary Table 3 provides a summary of the coding tree for the barriers to linkage to care and treatment.

#### Workplace related barriers

**Inflexible work schedules**. Some of the participants decried the strict nature of their work schedules, which did not allow them any time to go and access health care. This participant shared:

*I failed to go to the hospital because honestly there is no time. You are working the dayshift here, and nightshift somewhere else, because the more shifts you work, the more money you get. I asked my manager if I could go for 2 h, and he said that I should find another guard to cover my shift. I have still failed*. (P08, 40 years)

**Mandatory work transfers**. Men employed in private security services are frequently transferred or deployed to different locations in the country. This was a challenge for some of the participants when it came to linkage to care at new facilities. This is highlighted below:

*I was working in Kampala for 6 months when I got HIV-positive results. Now I have been transferred to*…. [another district] *and when I went to the hospital, it was overly complicated, and I had to start everything afresh*. (P06, 35 years)

#### Socio-economic barriers

**Far distance to health facilities from workplaces**. Some of the participants opted to link to care at health facilities close to their permanent homes. Unfortunately, these homes were far from the workplaces where they undertook HIVST, this, therefore, made it inconvenient for them to access treatment and care. One participant stated:

*Some of the health facilities are far from our workplaces, so we often must pay a lot of money to go there. This is inconvenient*. (P05, 35 years)

**Disruptive effects of the COVID-19 pandemic**. Several men did not link to treatment due to some of the unforeseen impacts of the COVID-19 pandemic:

*COVID-19 also was the main reason why I could not get treatment at that time because travel was restricted, so there was no way I could go for the treatment. However, by the time the lockdown restrictions were lifted, I was beginning to have doubts and up to now, I have not yet gone*. (P04, 35 years)

#### Health facility-related barriers

**Fear of stigma at the health facility**. Fear of experiencing stigmatizing behavior was another major barrier for some of the participants:

*There is stigma at the health facility. We go to a clinic where the company pays for our treatment. When you go there, everybody knows you and you just feel like everyone is looking at you*. (P01, 20 years)

**Lack of a centralized HIV care information management system**. Some participants expressed dissatisfaction with the way they are handled at health facilities when they desire to transfer their care. One participant stated:

*Every time I go to a new hospital, I must give all my information afresh and sometimes the health workers at the hospital do not understand but just send me away immediately. They tell you to go back to where you registered for treatment* [ART]. *They should organize a system whereby every time someone goes to any hospital their information is accessible*. (P09, 46 years)

#### Personal/individual factors

**Denial of HIV-positive results**. Some men were in denial of their HIV-reactive results, which hindered them from seeking healthcare because they did not believe the test results. A participant narrated:

*I cannot believe that those are my results. I have been living very well, how can these be my results? I will take another test after maybe 6 months with the blood test and then I can confirm. Why should I start treatment for a condition which I do not have?* (P04, 35 years)

## Discussion

This study explored the motivators for and barriers to linkage to HIV care and treatment among men who received reactive (positive) self-test results following workplace-based HIVST. Three categories emerged for the motivators, these were: consistent follow-up, enabling health facility environment and psychosocial support. The commonly reported specific motivators were mobile phone support, use of a linkage plan, referral forms, employing staff from the ART clinics and support from the employers. The recurring barriers to linking to care and treatment included worry about stigma at health facilities, inflexible work schedules, far distances to travel to access care and ART, and the negative effects of the COVID-19 pandemic.

Many men in the study reported that the mobile phone support they received after testing, greatly influenced their decision to link to treatment and care, which agrees with findings from other studies on HIVST (26–28). The support was in the form of phone calls, and SMS (Short Message Service) reminders, while for others it entailed social media support using smartphone applications such as WhatsApp. The increased access to mobile technologies presents an unprecedented opportunity to develop different mobile health (mHealth) interventions that may facilitate individuals' linkage to care following community-based HIV testing and HIVST interventions (29). The use of mobile technologies may be viable in settings like Uganda where there are over 24 million cellular phone subscribers (30). Existing evidence indicates that mHealth programs have taken advantage of the wide phone network coverage to enhance the gamut of HIV care ranging from HIV testing and identifying people who test HIV positive, to retention in care and adherence to HIV treatment (31–34). Unfortunately, the shortage of staff in some contexts may make it difficult for each tester to be followed-up for linkage to care. Additionally, it may be difficult to implement new programs that increase the workload of already overwhelmed staff in health facilities. Posadzki et al., suggest that automated systems can transmit messages, retrieve any required health data from patients, and maybe be a good substitute for face-to-face contact (35). Therefore, the limited resources can then be directed to persons living with HIV (PLWH) who request a callback, are unreachable or do not link to care. This calls for creative and affordable solutions that will not place added strain on the current staff.

Several men appreciated the creation of a linkage plan during the pre-test counseling session. The individualized linkage plan included five major aspects: (i) a choice of three facilities where the men could go immediately following an HIVST, (ii) a disclosure list (a list of people to whom the tester would wish to disclose his results), (iii) the option of participating in a peer support group, (iv) family support to link to care and (v) the choice of the mode of follow-up namely, phone calls, text messaging, or smartphone applications like WhatsApp or Facebook. While the initial planning was time-consuming, the men who did not anticipate a positive test result, found the plan particularly helpful as it gave them a semblance of control and direction. There is evidence that denial of one's HIV-positive diagnosis is a barrier to linkage to care (36, 37). Previous studies also suggest that having a prior plan facilitated disclosure of HIV-positive status (38, 39). Therefore, creating a linkage plan before taking the test may be useful in providing direction, and enhancing emotional readiness to accept a positive HIV diagnosis and seek further care.

Referral forms, slips, cards, or vouchers have been reported several times in the literature as a strong enabler of linkage to HIV care (37, 40, 41). In this study, we designed the study referral forms to collect the exact information that is recorded during standard HIV testing services. The participants were asked to present these forms to the health workers at the health facility if required. This was in addition to the linkage by the study team that worked at the health facility. This had the added benefit of helping the participants to quickly link to the health facilities in the study catchment area and made the transition seamless. As most of the participants were concerned about spending a lot of time at the health facility, the referral form greatly reduced their waiting time. To prevent data leaks, we used participant identity numbers and did not include the names, phone numbers or addresses on the referral forms. This had been agreed upon in a prior arrangement with the health facilities.

In South Africa, a study reported that several clients did not link to care because of previous unpleasant experiences at health facilities such as the long waiting time, poor treatment, and unprofessional conduct from health workers (36). Osingada et al., in their study about engaging men in Uganda, reported that they preferred to receive HIV services from distant health facilities because they did not trust the health workers whom they knew from their communities and were concerned about potential breaches of confidentiality (42). On the contrary, in this study, some of the men found it easier to link to care when they found familiar health workers at the health facilities, however, it is not clear whether this would still be the case if the health workers resided in the same communities as the participants. In this study, health workers from nearby health facilities were included as part of the study research team. This was one of the strategies to make a linkage to the health facility much easier for the participants. Initially, some of the men were concerned about the stigma at the health facilities, but the presence of a health worker they trusted helped them to navigate the health facility environment and lessened their concerns.

Previous studies have reported that top management support is a crucial element, for the success of any program related to HIV in the workplace (43–45). In this study, participants at some private security companies did not link to care because of their inflexible work schedules, and their inability to get time off to go for treatment and care. On the contrary, participants at other companies reported that they were able to get some time off to attend HIV clinics because the employers participated in the HIV testing program. In other places, the employers provided funding support for clinic visits. Furthermore, the support of the employer helped to mitigate stigma in the workplace. Therefore, employers are strongly encouraged to participate in HIV workplace initiatives, to improve linkage to treatment and care.

The current mitigation measures against COVID-19 transmission have increased the barriers to access to HIV services in Uganda. For example, a study among clinic-enrolled HIV-infected adults in Uganda found that 76% of them had their clinic attendance impacted by COVID-19. They highlighted challenges such as lack of transportation, police violence while enforcing the lockdown, and insufficient money for transportation (46). These findings resonate with ours, where some participants were unable to access HIV treatment due to the mandatory lockdowns and difficulty in accessing health facilities. This was coupled with the speed at which the pandemic escalated, which did not give enough time for the health system to adopt alternative measures to ensure access to essential medicines like ART, or treatment for TB. Amimo and colleagues (47) suggest that these restrictions could force the use of substandard drugs and/or doses, and lead to poor HIV and AIDS treatment outcomes, resulting in increased resistance to treatment. This strongly underscores the need for preparation and planning for future unexpected circumstances. Furthermore, programs should design contingency plans to ensure uninterrupted HIV care and treatment for PLWH. This agrees with the assertion (48) calling for the development of medium- and long-term policy-level and operational strategies for HIV care in the face of a potentially protracted COVID-19 pandemic, but also to prevent future shocks.

Several participants did not link to care or were not retained in care because of the challenges they faced while trying to transfer their care from one health facility to another. They were frustrated and recommended the introduction of a centralized HIV care information management system, which allows PLWH to access their care anywhere in the country. In South Africa, one of the proposed ways to resolve this is the use of a National identification, with each person in the country bearing a unique identifier (31). In that case, one may access HIV care and services anywhere in the country. While this seems feasible, it also raises concerns about potentially breaching patient confidentiality. In Uganda, Chamie et al. (49) used fingerprint biometric measurements for identification and confidentiality, during community-based HIV testing. However, further studies are needed to explore the potential users' acceptability of these proposed options.

### Study strengths and limitations

This is the first qualitative study to report the perspectives and user preferences of men who returned reactive HIV self-test results regarding linkage to care and treatment following workplace-based HIVST. One limitation was the use of phone interviews for data collection, which made it impossible to observe non-verbal cues from participants during the interview. Additionally, the COVID-19 restrictions and lockdowns at the time did not allow us to understand some of the naturally occurring wider structural challenges in the men's lives, because it was an extraordinary situation (50). Additionally, the study did not include a cost-effective analysis of the strategies that facilitated linkage to care to help policymakers in decision making, this should be the next step.

## Conclusion

The findings suggest the need for continual follow-up and open communication with individuals that test positive following workplace-based HIVST. This open communication and support may facilitate linkage to HIV care and treatment. Unfortunately, the limited health workforce in low-resource settings would hinder the use of strategies like constant provider-initiated follow-ups. There is an unprecedented opportunity to design mHealth interventions with automated or interactive voice responses that can provide reminders and follow-up individuals with positive self-test results. We also suggest continuing with tried and tested methods such as referral forms. Additionally, initiating individualized linkage plans during pre-test counseling and working in collaboration with HIV clinics may improve linkage to care about community-based HIVST. Furthermore, there is a need to put in place contingency plans for the continuity of HIV services in the event of future disasters or pandemics. Finally, the development of a national HIV care information management system is recommended. Thus, further research is needed to determine more innovative ways of implementing some of these methods without increasing the workload of current staff.

## Data availability statement

The raw data supporting the conclusions of this article will be made available by the authors, without undue reservation.

## Ethics statement

The studies involving human participants were reviewed and approved by Makerere University School of Health Sciences Research Ethics Committee (SHS-REC) (Ref. 2018-054) and the Uganda National Council for Science and Technology (UNCST) (Ref. HS 2672). The patients/participants provided their written informed consent to participate in this study.

## Author contributions

PM, NS, NK, and LN made substantial contributions to the conception of the project. TN, PM, and CO drafted the paper. NS, NK, TN, LN, and CO critically revised the manuscript for important intellectual content. All authors gave final approval for the work to be published and agree to be accountable for all aspects of the work in ensuring that questions related to the accuracy or integrity of any part of the work are appropriately investigated and resolved.

## Funding

This study and the WISe-Men Trial were supported by a NURTURE fellowship award funded under Grant Number D43TW010132 (PI: NS) from the Fogarty International Center of the National Institutes of Health. The funders played no role in study design and will play no role in data collection and analysis, the decision to publish, or the preparation of the manuscript. The contents are solely the responsibility of the authors and do not necessarily represent the official views of the supporting institutions.

## Conflict of interest

The authors declare that the research was conducted in the absence of any commercial or financial relationships that could be construed as a potential conflict of interest.

## Publisher's note

All claims expressed in this article are solely those of the authors and do not necessarily represent those of their affiliated organizations, or those of the publisher, the editors and the reviewers. Any product that may be evaluated in this article, or claim that may be made by its manufacturer, is not guaranteed or endorsed by the publisher.

## References

[1] UNAIDS. AIDSinfo. (2019). Available online at: http://aidsinfo.unaids.org/ (accessed July 15, 2020).

[2] UNAIDS. Blind spot- Reaching out to men and boys. (2017). Available online at: https://www.unaids.org/sites/default/files/media_asset/blind_spot_en.pdf (accessed November 20, 2020).

[3] UNAIDS. Country factsheets, Uganda 2019, HIV and AIDS Estimates (2019). Available online at: https://www.unaids.org/en/regionscountries/countries/uganda (accessed August 5, 2020).

[4] Figueroa C Johnson C Ford N Sands A Dalal S Meurant R. Reliability of HIV rapid diagnostic tests for self-testing compared with testing by health-care workers: a systematic review and meta-analysis. Lancet HIV. (2018) 5:e277–e90. 10.1016/S2352-3018(18)30044-4 29703707 PMC5986793

[5] WHO. HIV Selt-Testing at the Workplace. Geneva: WHO (2018).

[6] van Rooyen H Tulloch O Mukoma W Makusha T Chepuka L Knight LC. What are the constraints and opportunities for HIVST scale-up in Africa? Evidence from Kenya, Malawi and South Africa. J Int AIDS Soc. (2015) 18:19445. 10.7448/IAS.18.1.19445 25797344 PMC4369555

[7] Jennings L Conserve DF Merrill J Kajula L Iwelunmor J Linnemayr S. Perceived cost advantages and disadvantages of purchasing HIV self-testing kits among Urban Tanzanian Men: an inductive content analysis. J AIDS Clin Res. (2017) 8, 725–825. 10.4172/2155-6113.1000725 29051841 PMC5645025

[8] Harichund C Moshabela M. Acceptability of HIV self-testing in Sub-Saharan Africa: scoping study. AIDS Behav. (2018) 22:560–8. 10.1007/s10461-017-1848-9 28699017 PMC5764831

[9] Makusha T Knight L Taegtmeyer M Tulloch O Davids A Lim J. HIV self-testing could “revolutionize testing in South Africa, but it has got to be done properly”: perceptions of key stakeholders. PLoS ONE. (2015) 10:e0122783. 10.1371/journal.pone.0122783 25826655 PMC4380342

[10] Njau B Ostermann J Brown D Mühlbacher A Reddy E Thielman N. testing preferences in Tanzania: a qualitative exploration of the importance of confidentiality, accessibility, and quality of service. BMC Public Health. (2014) 14:838. 10.1186/1471-2458-14-838 25124140 PMC4141951

[11] Majam M. Progress on the scaling up of HIV testing in South Africa through varied distribution models using the oral HIV self-test kit. Oral Dis. (2020) 26 Suppl 1:137–40. 10.1111/odi.13395 32862537

[12] Njau B Covin C Lisasi E Damian D Mushi D Boulle A. A systematic review of qualitative evidence on factors enabling and deterring uptake of HIV self-testing in Africa. BMC Public Health. (2019) 19:1289. 10.1186/s12889-019-7685-1 31615461 PMC6794839

[13] Shapiro AE van Heerden A Krows M Sausi K Sithole N Schaafsma TT. An implementation study of oral and blood-based HIV self-testing and linkage to care among men in rural and peri-urban KwaZulu-Natal, South Africa. J Int AIDS Soc. (2020) 23 Suppl 2:e25514. 10.1002/jia2.25514 32589337 PMC7319114

[14] Hatzold K Gudukeya S Mutseta MN Chilongosi R Nalubamba M Nkhoma C. HIV self-testing: breaking the barriers to uptake of testing among men and adolescents in Sub-Saharan Africa, experiences from STAR demonstration projects in Malawi, Zambia and Zimbabwe. J Int AIDS Soc. (2019) 22 Suppl 1:e25244. 10.1002/jia2.25244 30907505 PMC6432104

[15] Amstutz A Lejone TI Khesa L Muhairwe J Bresser M Vanobberghen F. Home-based oral self-testing for absent and declining individuals during a door-to-door HIV testing campaign in rural Lesotho (HOSENG): a cluster-randomised trial. Lancet HIV. (2020). 10.1016/S2352-3018(20)30233-2 33045193

[16] Zhang C Koniak-Griffin D Qian HZ Goldsamt LA Wang H Brecht ML. Impact of providing free HIV self-testing kits on frequency of testing among men who have sex with men and their sexual partners in China: a randomized controlled trial. PLoS Med. (2020) 17:e1003365. 10.1371/journal.pmed.1003365 33035206 PMC7546488

[17] Choko AT Corbett EL Stallard N Maheswaran H Lepine A Johnson CC. HIV self-testing alone or with additional interventions, including financial incentives, and linkage to care or prevention among male partners of antenatal care clinic attendees in Malawi: An adaptive multi-arm, multi-stage cluster randomised trial. PLoS Med. (2019) 16:e1002719. 10.1371/journal.pmed.1002719 30601823 PMC6314606

[18] Neuman M Taegtmeyer M Hatzold K Johnson CC Weiss HA Fielding K. Challenges in measurement of linkage following HIV self-testing: examples from the STAR Project. J Int AIDS Soc. (2019) 22 Suppl 1:e25238. 10.1002/jia2.25238 30907491 PMC6432105

[19] Muwanguzi P. Workplace-based HIV Self-testing among men in Uganda (WISe-Men) ClinicalTrials.gov: U.S. National Library of Medicine (2019). Available from: https://ClinicalTrials.gov/show/NCT04164433 (updated September 16, 2020).

[20] Muwanguzi PA Ngabirano TD Kiwanuka N Nelson LE Nasuuna EM Osingada CP. The effects of workplace-based HIV self-testing on uptake of testing and linkage to HIV Care or prevention by men in Uganda (WISe-Men): protocol for a cluster randomized trial. JMIR Res Protoc. (2021) 10:e25099. 10.2196/25099 34723826 PMC8593794

[21] GOU. COVID-19 Response info hub-Timeline. (2020). Available online at: https://covid19.gou.go.ug/timeline.html (accessed November 21, 2020).

[22] Elo S Kyngas H. The qualitative content analysis process. J Adv Nurs. (2008) 62:107–15. 10.1111/j.1365-2648.2007.04569.x 18352969

[23] Lincoln YS Guba EG. But is it rigorous? Trustworthiness and authenticity in naturalistic evaluation. New Direct Program Eval. (1986) 1986:73–84. 10.1002/ev.1427

[24] Bradshaw C Atkinson S Doody O. Employing a qualitative description approach in health care research. Global Qual Nursing Res. (2017) 4:2333393617742282. 10.1177/2333393617742282 29204457 PMC5703087

[25] Tong A Sainsbury P Craig J. Consolidated criteria for reporting qualitative research (COREQ): a 32-item checklist for interviews and focus groups. Int J Qual Health Care. (2007) 19:349–57. 10.1093/intqhc/mzm042 17872937

[26] Mokgatle MM Madiba S. High Acceptability of HIV Self-testing among technical vocational education and training college students in Gauteng and North West Province: What are the implications for the scale up in South Africa? PLoS ONE. (2017) 12:e0169765. 10.1371/journal.pone.0169765 28141858 PMC5283675

[27] Choko AT Kumwenda MK Johnson CC Sakala DW Chikalipo MC Fielding K. Acceptability of woman-delivered HIV self-testing to the male partner, and additional interventions: a qualitative study of antenatal care participants in Malawi. J Int AIDS Soc. (2017) 20:21610. 10.7448/IAS.20.1.21610 28691442 PMC5515040

[28] Martínez Pérez G Cox V Ellman T Moore A Patten G Shroufi A. 'I Know that I Do Have HIV but Nobody Saw Me': Oral HIV Self-Testing in an Informal Settlement in South Africa. PLoS ONE. (2016) 11:e0152653. 10.1371/journal.pone.0152653 27044006 PMC4820175

[29] Forrest JI Wiens M Kanters S Nsanzimana S Lester RT Mills EJ. Mobile health applications for HIV prevention and care in Africa. Curr Opin HIV AIDS. (2015) 10:464–71. 10.1097/COH.0000000000000198 26352394

[30] UBOS. Uganda Bureau Of Statistics. (2019) statistical abstract.

[31] Comulada WS Wynn A van Rooyen H Barnabas RV Eashwari R van Heerden A. Using mHealth to deliver a home-based testing and counseling program to improve linkage to care and ART adherence in rural South Africa. Prev Sci. (2019) 20:126–36. 10.1007/s11121-018-0950-1 30259235 PMC6358509

[32] Siedner MJ Santorino D Haberer JE Bangsberg DR. Know your audience: predictors of success for a patient-centered texting app to augment linkage to HIV care in rural Uganda. J Med Internet Res. (2015) 17:e78. 10.2196/jmir.3859 25831269 PMC4389108

[33] Palmer MJ Henschke N Villanueva G Maayan N Bergman H Glenton C. Targeted client communication via mobile devices for improving sexual and reproductive health. Cochrane Database Syst Rev. (2020) 8:Cd013680. 10.1002/14651858.CD013680 32779730 PMC8409381

[34] Dillingham R Ingersoll K Flickinger TE Waldman AL Grabowski M Laurence C. PositiveLinks: a mobile health intervention for retention in HIV care and clinical outcomes with 12-month follow-up. AIDS Patient Care STDS. (2018) 32:241–50. 10.1089/apc.2017.0303 29851504 PMC5982157

[35] Posadzki P Mastellos N Ryan R Gunn LH Felix LM Pappas Y. Automated telephone communication systems for preventive healthcare and management of long-term conditions. Cochrane Database Syst Rev. (2016) 12:Cd009921. 10.1002/14651858.CD009921.pub2 27960229 PMC6463821

[36] Naik R Zembe W Adigun F Jackson E Tabana H Jackson D. What influences linkage to care after home-based HIV counseling and testing? AIDS Behav. (2018) 22:722–32. 10.1007/s10461-017-1830-6 28643242 PMC5847222

[37] Kayabu DE Ngocho JS Mmbaga BT. Effective linkage from point of HIV testing to care and treatment in Tanga region, Tanzania. PLoS ONE. (2018) 13:e0201644. 10.1371/journal.pone.0201644 30114244 PMC6095519

[38] Kiula ES Damian DJ Msuya SE. Predictors of HIV serostatus disclosure to partners among HIV-positive pregnant women in Morogoro, Tanzania. BMC Public Health. (2013) 13:433. 10.1186/1471-2458-13-433 23641927 PMC3668140

[39] Qiao S Li X Stanton B. Disclosure of parental HIV infection to children: a systematic review of global literature. AIDS Behav. (2013) 17:369–89. 10.1007/s10461-011-0069-x 22016331 PMC6234003

[40] Choko AT MacPherson P Webb EL Willey BA Feasy H Sambakunsi R. Uptake, accuracy, safety, and linkage into care over 2 years of promoting annual self-testing for HIV in Blantyre, Malawi: a community-based prospective study. PLoS Med. (2015) 12:e1001873. 10.1371/journal.pmed.1001873 26348035 PMC4562710

[41] Sanga ES Mukumbang FC Mushi AK Olomi W Lerebo W Zarowsky C. Processes and dynamics of linkage to care from mobile/outreach and facility-based HIV testing models in hard-to-reach settings in rural Tanzania. Qual Find Mixed Methods Study AIDS Res Ther. (2018) 15:21. 10.1186/s12981-018-0209-8 30458874 PMC6247671

[42] Osingada CP Siu G Amollo M Muwanguzi P Sewankambo N Kiwanuka N. Acceptability of HIV testing for men attending televised football venues in Uganda. BMC Public Health. (2019) 19:1136. 10.1186/s12889-019-7478-6 31426776 PMC6700992

[43] Chatora B Chibanda H Kampata L Wilbroad M. HIV/AIDS workplace policy addressing epidemic drivers through workplace programs. BMC Public Health. (2018) 18:180. 10.1186/s12889-018-5072-y 29370779 PMC5785818

[44] Knoblauch AM Divall MJ Owuor M Nduna K Ng'uni H Musunka G. Experience and lessons from health impact assessment guiding prevention and control of HIV/AIDS in a copper mine project, northwestern. Zambia Infect Dis Poverty. (2017) 6:114. 10.1186/s40249-017-0320-4 28673329 PMC5496403

[45] Arimoto Y Hori N Ito S Kudo Y Tsukada K. Impacts of an HIV Counselling and testing initiative: results from an experimental intervention in South Africa. (2012).

[46] Linnemayr S Jennings Mayo-Wilson L Saya U Wagner Z MacCarthy S Walukaga S. HIV Care Experiences During the COVID-19 Pandemic: Mixed-Methods Telephone Interviews with Clinic-Enrolled HIV-Infected Adults in Uganda. AIDS Behav. (2020). 10.1007/s10461-020-03032-8 32918641 PMC7486807

[47] Amimo F Lambert B Magit A. What does the COVID-19 pandemic mean for HIV, tuberculosis, and malaria control? Trop Med Health. (2020) 48:32. 10.1186/s41182-020-00219-6 32425653 PMC7218555

[48] Rewari BB Mangadan-Konath N Sharma M. Impact of COVID-19 on the global supply chain of antiretroviral drugs: a rapid survey of Indian manufacturers. WHO South East Asia J Public Health. (2020) 9:126–33. 10.4103/2224-3151.294306 32978345

[49] Chamie G Kwarisiima D Clark TD Kabami J Jain V Geng E. Uptake of community-based HIV testing during a multi-disease health campaign in rural Uganda. PLoS ONE. (2014) 9:e84317. 10.1371/journal.pone.0084317 24392124 PMC3879307

[50] Muwanguzi PA Kutyabami P Osingada CP Nasuuna EM Kitutu FE Ngabirano TD. Conducting an ongoing HIV clinical trial during the COVID-19 pandemic in Uganda: a qualitative study of research team and participants' experiences and lessons learnt. BMJ Open. (2021) 11:e048825. 10.1136/bmjopen-2021-048825 33883157 PMC8061567

