# Supplementary material for: Linkage to care and treatment among men with reactive HIV self-tests after workplace-based testing in Uganda: A qualitative study
Source: Front Public Health. 2022 Oct 12;10:650719. doi: 10.3389/fpubh.2022.650719 (PMC9597242; doi:10.3389/fpubh.2022.650719)
Supplement: Supplementary file 1 [file Data_Sheet_1.docx]

**Phone interview**

**Before commencing the interview, ensure written consent is received. See consent form**

1. Can you please describe your experience of HIV self-testing?
2. What was your experience of taking an HIV test at the workplace?
3. What did you do after you received your positive test results? To whom did you disclose these results?
4. How long after receiving the test results did you go to the hospital? What motivated you to go to the hospital at that time?
5. How easy/difficult was it for you to receive care at the hospital? What made it easy or difficult?
6. Was there anything special that encouraged you to go to the hospital?
7. Was there anything that discouraged you from going to the hospital after the positive test results? Would you still like to go to the hospital? How would you like to be supported?
8. Have you started the treatment?
   1. If yes, What treatment are you taking? Are you still taking your medication? How are you taking it?
   2. If no, why have you not started the treatment? Is there anything specific that prevented you from starting the treatment
9. Is there anything memorable from your experience of self-testing to accessing care at the hospital? What stood out for you?
10. Is there anything that could have been done better to your experience of self-testing and accessing care at the hospital?

*Thank you for your time and participation, we have learnt a lot from our discussion here today and we hope the time has also been useful to you.*

**Table 1: Demographics of study participants**

| **Participant Characteristics (*N* =12)** | **Frequency**  **(*n*)** | **Percentage (%)** |
| --- | --- | --- |
| **Age range, years** |  |  |
| 18-25 | 2 | 16.7 |
| 26-35 | 4 | 33.3 |
| 36-45 | 3 | 25.0 |
| 46-60 | 3 | 25.0 |
| **Marital status** |  |  |
| Married | 8 | 66.7 |
| Unmarried | 1 | 8.3 |
| Divorced/separated | 3 | 25.0 |
| **Highest education level attained** |  |  |
| Primary | 3 | 25.0 |
| Secondary | 7 | 58.3 |
| Tertiary | 2 | 16.7 |

**Table 2: Coding tree for motivators for linkage to care following reactive HIV self-test results**

| **Theme** | **Category** | **Sub-category** |  |
| --- | --- | --- | --- |
| Motivators for linkage to care and treatment | Communication | Phone reminders |  |
|  |  | Open channels of communication |  |
|  |  | Consistent and regular follow-up |  |
|  | Navigating health facility systems and processes | Easy access to care |  |
|  |  | Enabling health facility environment |  |
|  |  | Inclusion of ART clinic staff among the research team |  |
|  |  | Trustworthiness of health workers |  |
|  | Linkage support | Individualized linkage plan |  |
|  |  | Linkage facilitation |  |
|  |  | Referral forms | |
|  |  | Pre-arranged clinic appointments |  |
|  |  | Study team members acting as linkage companions |  |
|  | Psychosocial support | Counselling sessions |  |
|  |  | Online and social media support |  |
|  |  | Family linkage support |  |
|  |  | Peer support |  |
|  | Workplace environment | Employers support |  |
|  |  | Workplace schedules and policies |  |

**Table 3: Coding tree for barriers to linkage to care following positive HIV self-test results**

|  | **Category** | **Sub-category** |
| --- | --- | --- |
| Barriers to linkage to care | Workplace related barriers | Inflexible work schedules |
|  |  | Mandatory work transfers |
|  | Socio-economic barriers | Far distance to health facilities |
|  |  | Disruptive effects of COVID-19 |
|  | Health facility related barriers | Fear of stigma at health facility |
|  |  | Lack of centralized HIV care Information Management system |
|  | Personal/individual factors | Denial of HIV results |
